# Association between Hyperglycemia at Hospital Presentation and Hospital Outcomes in COVID-19 Patients with and without Type 2 Diabetes: A Retrospective Cohort Study of Hospitalized Inner-City COVID-19 Patients

**DOI:** 10.3390/nu13072199

**Published:** 2021-06-26

**Authors:** Nipith Charoenngam, Sara M. Alexanian, Caroline M. Apovian, Michael F. Holick

**Affiliations:** 1 Section Endocrinology, Diabetes, Nutrition and Weight Management, Department of Medicine, Boston University School of Medicine, Boston, MA 02118, USA; ncharoen@bu.edu (N.C.); sara.alexanian@bmc.org (S.M.A.); 2 Department of Medicine, Faculty of Medicine Siriraj Hospital, Mahidol University, Bangkok 10700, Thailand; 3 Section of Endocrinology, Diabetes and Hypertension, Brigham and Women’s Hospital, Harvard Medical School, Boston, MA 02115, USA; capovian@partners.org

**Keywords:** COVID-19, SARS-CoV-2, coronavirus, mortality, blood glucose, hyperglycemia, type 2 diabetes mellitus

## Abstract

This study aimed to determine the relationships among hyperglycemia (HG), the presence of type 2 diabetes (T2D), and the outcomes of COVID-19. Demographic data, blood glucose levels (BG) measured on admission, and hospital outcomes of COVID-19 patients hospitalized at Boston University Medical Center from 1 March to 4 August 2020 were extracted from the hospital database. HG was defined as BG > 200 mg/dL. Patients with type 1 diabetes or BG < 70 mg/dL were excluded. A total of 458 patients with T2D and 976 patients without T2D were included in the study. The mean ± SD age was 56 ± 17 years and 642 (45%) were female. HG occurred in 193 (42%) and 42 (4%) of patients with and without T2D, respectively. Overall, the in-hospital mortality rate was 9%. Among patients without T2D, HG was statistically significantly associated with mortality, ICU admission, intubation, acute kidney injury, and severe sepsis/septic shock, after adjusting for potential confounders (*p* < 0.05). However, only ICU admission and acute kidney injury were associated with HG among patients with T2D (*p* < 0.05). Among the 235 patients with HG, the presence of T2D was associated with decreased odds of mortality, ICU admission, intubation, and severe sepsis/septic shock, after adjusting for potential confounders, including BG (*p* < 0.05). In conclusion, HG in the subset of patients without T2D could be a strong indicator of high inflammatory burden, leading to a higher risk of severe COVID-19.

## 1. Introduction

Severe acute respiratory syndrome coronavirus 2 (SARS-CoV-2) is the novel strain of coronavirus that causes the coronavirus disease (COVID-19). Due to its infectivity and transmissibility, this emerging infectious disease quickly became a pandemic, leading to global economic and social disruption [1,2]. Most previously healthy patients who contract COVID-19 are asymptomatic or develop only mild respiratory symptoms. However, a significant number of patients who have pre-existing medical conditions may develop severe complications that result in morbidity and mortality, such as acute respiratory distress syndrome (ARDS), vascular thrombosis, septic shock, and multi-organ dysfunction [3,4].

Some studies have shown that patients with type 2 diabetes (T2D) have an increased risk of morbidity and mortality in the setting of COVID-19 infection [5,6,7]. This is thought to be primarily mediated by the alteration of the immune system and renin-angiotensin-aldosterone systems, endothelial dysfunction, and oxidative stress due to chronic hyperglycemia [8,9]. In addition, recent evidence has highlighted not only that the severe systemic inflammation seen in COVID-19 patients causes stress-induced hyperglycemia, but also that SARS-CoV-2 can directly infect the pancreatic β-cells, resulting in β-cell dysfunction and insulin deficiency [9,10]. This suggests that the association between hyperglycemia and COVID-19 severity could be bidirectional. 

A growing number of observational studies have shown that hyperglycemia is a strong predictor of morbidity and mortality in hospitalized and critically ill COVID-19 patients, independent of pre-existing diabetes status [11,12,13]. However, it is still unclear how the combination of underlying T2D and the presence of hyperglycemia at the time of hospital admission is associated with the inflammatory burden and outcomes of COVID-19. Therefore, the primary objective of this study is to investigate the association of hyperglycemia based on blood glucose (BG) upon hospital admission with hospital outcomes and inflammatory markers among COVID-19 patients with and without T2D. Furthermore, we aimed to investigate whether the presence of T2D among patients with hyperglycemia was associated with these outcomes after controlling for BG on admission, along with other potential confounding variables.

## 2. Materials and Methods

### 2.1. Study Population

This is a retrospective chart review cohort study in adult patients aged ≥ 18 years old who were COVID-19-positive and hospitalized at Boston University Medical Center from 1 March to 4 August 2020. All patients who tested positive for SARS-CoV-2 nucleic acid testing and had BG levels measured on hospital admission were included in the study. Patients with type 1 diabetes and BG levels of <70 mg/dL were excluded from the analysis. The study protocol was approved by the Boston University Medical Campus Institutional Review Board (H-40341).

### 2.2. Study Measurements

We extracted the characteristics of the patients from the Boston University Medical Center hospital database. These included age; sex; race; latest body mass index (BMI); self-reported history of smoking; alcohol use; and underlying comorbidities determined by the International Classification of Disease, 10^th^ Revision, Clinical Modification (ICD-10-CM). Diagnosis with the following underlying comorbidities was recorded: T2D (E11), T1D (E10), hypertension (I10), dyslipidemia (E78), coronary heart disease (I25), cerebrovascular disease (I66, I67, I69, G45), chronic obstructive pulmonary disease (COPD, J44), asthma (J45), chronic kidney disease (CKD, N18), end-stage renal disease (ESRD, N18.6), malignancy (I50), human immunodeficiency virus infection (B20), and heart failure (I50).

We also recorded the most recent hemoglobin A1C (HbA1C) levels prior to admission within the past 2 years, the receipt of antidiabetic medications prior to admission, and in-hospital medical therapy for COVID-19 (i.e., azithromycin, hydroxychloroquine, colchicine, corticosteroids, interleukin-6 antibodies, and interleukin-1 receptor antagonists).

Biochemical data, including BG levels measured at the time of hospitalization and most recent clinic estimated glomerular filtration rate (eGFR), were extracted from the hospital database. Inflammatory marker levels at the time of hospitalization or as soon thereafter as possible (within 48 h after admission) were also extracted. These included plasma C-reactive protein (CRP), plasma D-dimer, erythrocyte sedimentation rate (ESR), plasma ferritin, and serum lactate dehydrogenase (LDH). Hyperglycemia was defined as a BG level of >200 mg/dL at hospital presentation in the emergency department or as soon as possible thereafter. BG was measured by either point-of-care testing or as serum glucose included in a basic or comprehensive metabolic panel.

The primary outcome of this study was in-hospital mortality. Secondary outcomes included intensive care unit (ICU) admission, need for intubation, hospital length of stay, inflammatory marker levels, and morbidity outcomes, including hospital diagnosis based on the ICD-10-CM codes of ARDS (J80), myocardial infarction (I21), acute kidney injury (N17), severe sepsis/septic shock (R65.20, R65-21), and diabetic ketoacidosis. All hospital outcomes were retrieved from the hospital database and were validated by manual chart review.

### 2.3. Statistical Analysis

Continuous normally distributed variables were reported as arithmetic means with standard deviations (SDs). Continuous non-normally distributed variables were reported as medians with interquartile range (IQR)s. Categorical variables were reported as number of patients with percentages. Patients were categorized into four groups, including (1) patients without T2D and with hyperglycemia, (2) patients without T2D and without hyperglycemia, (3) patients with T2D and with hyperglycemia, and (4) patients with T2D and without hyperglycemia. Univariate comparisons of baseline characteristics and outcomes among groups were performed using the independent sample *t*-test for normally distributed continuous data, the Mann–Whitney-U test for non-normally distributed continuous data, and the Chi-square or Fischer’s exact test for categorical data. 

Comparisons of inflammatory marker levels among groups were analyzed using the analysis of variance followed by Bonferroni’s post hoc test for pairwise comparison (for normally distributed data) and the Kruskal–Wallis test followed by Dunn’s test for pairwise comparison (for non-normally distributed data). These data were presented as box plots. 

Multivariate logistic regression was used to determine odds ratios (OR) and 95% confidence intervals (CIs) were used to compare hospital outcomes between patients with and without hyperglycemia among patients with and without T2D. These models were adjusted for potential confounding variables, including age, sex, body mass index, smoking, alcohol drinking, and underlying comorbidities. Additionally, multivariate logistic regression was used to compare hospital outcomes between patients with and without T2D among patients with hyperglycemia. These models were adjusted for BG at, hospital admission in addition to age, sex, body mass index, smoking, alcohol drinking and the aforementioned underlying comorbidities.

To further investigate the association of glycemic control and the use of antidiabetic medications with hospital outcomes, univariate and/or multivariate comparisons between T2D patients with HbA1C < 7% and ≥7% and between T2D patients with and without the use of each antidiabetic medication were performed. 

## 3. Results

A total of 1478 COVID-19 patients were hospitalized at the Boston University Medical Center from 1 March 2020 to 4 August 2020. Among them, eight patients with type 1 diabetes, 21 patients with BG < 70 mg/dL, and 15 patients with unavailable BG levels were excluded from the study. Finally, 1434 patients, including 458 patients with T2D and 976 patients without diabetes, were found to fulfill the eligibility criteria and were included in the analysis. The median (IQR) age was 51.0 (44.0–68.0) years and 642 (44.8%) were female. The characteristics of patients with and without T2D and with hyperglycemia (BG > 200 mg/dL) and without hyperglycemia (BG ≤ 200 mg/dL) at hospital presentation are demonstrated in Table 1. A total of 193 (19.8%) patients with T2D had hyperglycemia (BG > 200 mg/dL), while 42 (4.3%) patients without T2D had hyperglycemia at hospital presentation. Among patients without T2D, those with hyperglycemia had lower rates of alcohol use history and treatment with hydroxychloroquine and higher frequency of use of corticosteroids and remdesivir (all *p* < 0.05). Among patients with T2D, those with hyperglycemia had poorer BG control based on the most recent HbA1C and a higher incidence of sulfonylurea use (both *p* < 0.005, Table 1). Moreover, they represented a lower proportion of females and had a lower frequency of smoking history, heart failure, cerebrovascular disease, COPD, CKD, ESRD, and malignancy (all *p* < 0.05, Table 1). Diabetic ketoacidosis occurred in 13 and 3 patients with and without a history of T2D, respectively. Among them, 8 patients were admitted to the ICU and 3 patients died during the hospitalization. All of them had a history of T2D.

### 3.1. Inflammatory Marker Levels

**Figure 1:**
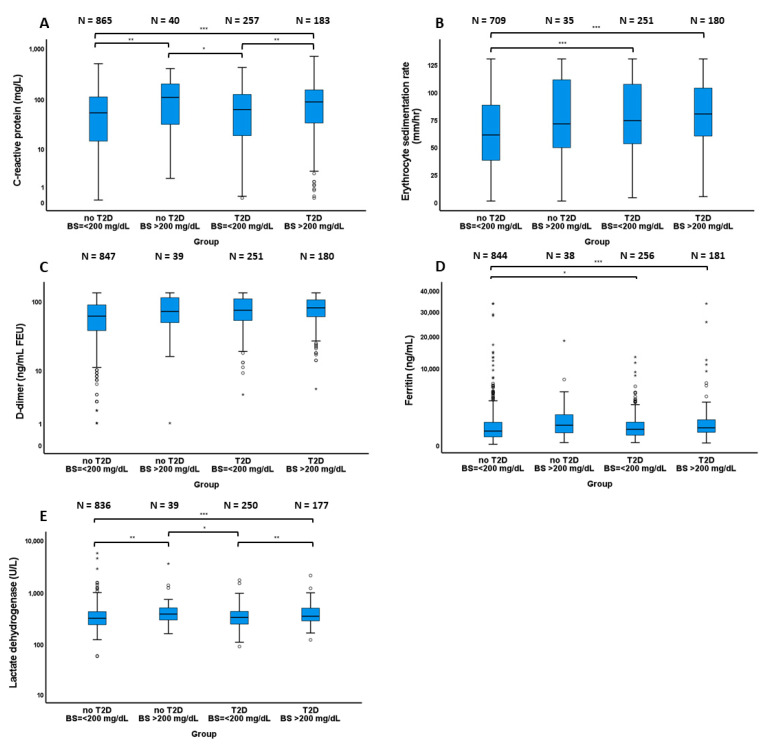
Comparisons of inflammatory markers among patients with and without type 2 diabetes who had admission blood glucose ≤200 and >200 mg/dL. (**A**) C-reactive protein (**B**) Erythrocyte sedimentation rate (**C**) D-dimer (**D**) Ferritin (**E**) Lactate dehydrogenase. “*” denotes *p* < 0.05; “**” denotes *p* < 0.01; “***” denotes *p* < 0.001.

Comparisons of inflammatory marker levels among the groups stratified by T2D and hyperglycemia status are demonstrated in Figure 1. Plasma CRP and serum LDH were statistically significantly higher in patients with hyperglycemia among both patients with and without T2D (all *p* < 0.01). Compared with patients with T2D without hyperglycemia, hyperglycemic patients without T2D had statistically significantly higher plasma CRP and serum LDH (both *p* < 0.05). Additionally, hyperglycemic patients with T2D had statistically significantly plasma CRP and serum LDH than patients without T2D who did not have hyperglycemia (both *p* < 0.001). In comparison with patients without T2D who did not have hyperglycemia, those with T2D with and without hyperglycemia both had statistically significantly higher ESR (both *p* < 0.001). Finally, patients without T2D who did not have hyperglycemia had statistically significantly lower plasma ferritin than the other groups (all *p* < 0.05). 

An additional analysis to determine the association between inflammatory marker levels and in-hospital mortality was performed. We found in the multivariate analysis that increased plasma CRP was statistically significantly associated with in-hospital mortality (adjusted OR 1.093, 95%CI 1.070–1.116 per 10 mg/L increase). The same association was observed in the model of ESR (adjusted OR 1.109, 95%CI 1.040–1.183 per 10 mm/h increase) and LDH (adjusted OR 1.025, 95%CI 1.016–1.033 per 10 U/L increase). Effect estimates were adjusted for age; sex; body mass index; smoking; alcohol drinking; and underlying comorbidities, including T2D, hypertension, dyslipidemia, coronary heart disease, cerebrovascular disease, COPD, asthma, CKD, ESRD, malignancy, HIV infection, and heart failure.

### 3.2. Univariate Comparisons between Patients with and without Hyperglycemia 

Hospital outcomes stratified by T2D and hyperglycemia at hospital presentation status are demonstrated in Table 2. Among patients with T2D, those with hyperglycemia had a statistically significantly higher rate of acute kidney injury than those without hyperglycemia (53.9% vs. 36.2%, *p* < 0.001). However, the in-hospital mortality and other clinical outcomes did not significantly differ across the groups. 

Among patients without T2D compared with those without hyperglycemia, patients with hyperglycemia had statistically significantly longer hospital stays (mean ± SD: 10.1 ± 16.8 vs. 7.1 ± 8.3 days, *p* = 0.036) and higher rates of in-hospital mortality (21.4% vs. 6.6%, *p* < 0.001), ICU admission (40.0% vs. 16.2%, *p* < 0.001), intubation (35.7% vs. 10.7%, *p* < 0.001), acute kidney injury (40.5% vs. 23.4%, *p* = 0.012), and severe sepsis/septic shock (23.8% vs. 7.8%, *p* < 0.001). 

### 3.3. Multivariate Comparisons between Patients with and without Hyperglycemia

The adjusted associations between hyperglycemia and hospital outcomes in subgroups of patients with and without T2D are demonstrated Table 3. Among patients with T2D, those with hyperglycemia had statistically significantly higher odds of ICU admission (adjusted OR 1.69, 95% CI, 1.03–2.79) and acute kidney injury (adjusted OR 2.45, 95%CI, 1.54–3.90). Among patients without T2D, those with hyperglycemia had statistically significantly higher odds of in-hospital mortality (adjusted OR 4.46, 95% CI, 1.77–11.20), ICU admission (adjusted OR 3.71, 95% CI, 1.84–7.48), intubation (adjusted OR 4.82, 95% CI, 2.39–4.72), acute kidney injury (adjusted OR 2.29, 95% CI, 1.14–4.60), and severe sepsis/septic shock (adjusted OR 4.14, 95% CI, 1.85–9.24). Effect estimates were adjusted for age; sex; body mass index; smoking; alcohol drinking; and underlying comorbidities, including hypertension, dyslipidemia, coronary heart disease, cerebrovascular disease, COPD, asthma, CKD, ESRD, malignancy, HIV infection, and heart failure.

### 3.4. Comparisons between Hyperglycemic Patients with and without Type 2 Diabetes

Univariate and multivariate comparisons between hyperglycemic patients with and without T2D are demonstrated in Table 4. In the univariate analysis, compared with hyperglycemic patients with T2D, hyperglycemic patients without T2D had a statistically significantly higher rate of intubation (35.7% vs. 17.6%, *p* = 0.009) and tended to have a higher rate of ICU admission (40% vs. 24.9%, *p* = 0.051). In the multivariate analysis, among patients with hyperglycemia patients without T2D had statistically significantly increased odds of in-hospital mortality (adjusted OR 5.05, 95% CI, 1.34–18.99), ICU admission (adjusted OR 2.68, 95% CI, 1.07–6.71), intubation (adjusted OR 3.78, 95% CI, 1.37–10.46), and severe sepsis/septic shock (adjusted OR 3.90, 95% CI, 1.28–11.99). Effect estimates were adjusted for BG at hospital presentation in addition to age, sex, body mass index, smoking, alcohol drinking, and underlying comorbidities.

### 3.5. Association of Long-Term Glycemic Control and Use of Anti-Diabetic Medication and Hospital Outcomes

Among patients with T2D, those with HbA1C ≥ 7% had a statistically significantly higher rate of acute kidney injury than those with HbA1C < 7% (51.9% vs. 41.1%, *p* = 0.050). However, the association was no longer statistically significant in the multivariate model after adjusting for age, sex, and the presence of chronic kidney disease (adjusted OR 1.26, 95%CI 0.77–2.07). Other hospital outcomes were not significantly different between the two groups stratified by HbA1C status. Additionally, a lower death rate was observed in metformin users (9.3% vs. 16.2%, *p* = 0.032) and sulfonylurea users (4.7% vs. 13.9%, *p* = 0.010) compared with those who did not use the medications. The association of HbA1C status and the use of anti-diabetic medications among patients with T2D is demonstrated in the Table S1.

### 3.6. Association between Pre-Existing Kidney Dysfunction and In-Hospital Mortality

After excluding 67 patients with ESRD, increased baseline eGFR was found to be a significant predictor of decreased in-hospital mortality (adjusted OR 0.865, 95%CI 0.800–0.935 per 10 mL/min/1.73 m^2^ increase). The association was statistically significant in both subgroups of patients with T2D (adjusted OR 0.853, 95%CI 0.750–0.970 per 10 mL/min/1.73 m^2^ increase) and without T2D (adjusted OR 0.856, 95%CI 0.774–0.940 per 10 mL/min/1.73 m^2^ increase). Effect estimates were adjusted for age; sex; body mass index; smoking; alcohol drinking; and underlying comorbidities, including T2D (for the analysis of all patients), hypertension, dyslipidemia, coronary heart disease, cerebrovascular disease, COPD, asthma, malignancy, HIV infection, and heart failure. 

## 4. Discussion

In this retrospective cohort study, we observed that hospitalized COVID-19 patients without T2D who had hyperglycemia defined by BG > 200 mg/dL upon hospital admission had significantly higher odds of in-hospital mortality and morbidity compared with patients without T2D who did not have hyperglycemia. On the other hand, only ICU admission and acute kidney injury were found to be significantly associated with hyperglycemia among T2D patients. Furthermore, we found that hyperglycemic patients in both subgroups with and without T2D had significantly higher levels of inflammatory markers upon admission (i.e., CRP and LDH), which were found to be associated with increased in-hospital mortality in our cohort as well as in other previous studies [14]. Interestingly, compared with hyperglycemic patients with T2D, those without T2D had an approximately 5-time higher odds of mortality. 

Our observation is in line with the result from the meta-analysis of 10 studies showing that hyperglycemia in patients without diabetes was associated with higher risks of critical illness (pooled OR 1.8, 95%CI 1.4–2.5) and mortality (pooled OR 2.8, 95%CI 1.6–5.0) [15]. Our results also support those of the previous studies showing that the association was stronger among patients without pre-existing diabetes than those with a history of diabetes [11,12].

It has been well-documented that hyperglycemia is associated with adverse clinical outcomes in various settings, such as critical illness and perioperative periods [16,17,18]. In the absence of pre-existing diabetes, hyperglycemia can develop secondary to physiologic stress, which causes increased sympathetic stimulation and a subsequent rise in circulating catecholamines, cortisol, glucagon, and growth hormone levels, thereby inducing hepatic gluconeogenesis [17,18]. In addition, systemic inflammation and increased proinflammatory cytokines (e.g., tumor necrosis factor-α, interleukin-1, and interleukin-6) can lead to impaired insulin signaling, thereby exacerbating hyperglycemia [19,20]. It is also worth noting that SARS-CoV-2 can infect the pancreatic β-cells, which results in β-cell dysfunction and insulin deficiency [9,10]. Therefore, patients with a high degree of viremia may develop new-onset type 1 diabetes or insulin deficiency and subsequent hyperglycemia emergencies, which could lead to significant morbidity and mortality [21,22,23]. This is supported by our data, revealing 16 patients (13 with pre-existing T2D and 3 without T2D) who developed diabetic ketoacidosis. The questions of whether or not and how likely this is to lead to new-onset diabetes as a long-term sequelae needs to be further investigated.

For the above reasons, hyperglycemia is believed to be a strong indicator of COVID-19 morbidity and mortality. It is of particular interest that there is stronger association between hyperglycemia and disease severity among patients without a history of T2D. In addition, patients with new-onset hyperglycemia were found to have an approximately 5 times higher risk of mortality and 2.5 times higher risk of ICU admission compared with hyperglycemic patients with T2D, even after adjusting for admission blood glucose. This is likely due to the fact that they suffered from a higher degree of physiologic stress and systemic inflammation than those with T2D, who may have developed hyperglycemia to the same level due to their baseline insulin resistance but with relatively milder COVID-19 disease severity. This observation may also suggest that the development of acute hyperglycemia plays a more important role than chronic insulin resistance in mediating the severity of COVID-19. It remains to be seen if inpatient strict BG control has a mortality benefit in COVID-19 hyperglycemic patients with or without T2D, given the evidence that insulin infusion is associated with a lower risk of severe COVID-19 [24] and that tight glycemic control could improve hospital outcomes in patients with acute coronary events [25,26]. 

It is worth noting that, among the patients with T2D in our study, patients without hyperglycemia tended to be older, were less likely to be female, and had higher frequencies of certain comorbidities. These factors are associated with frailty and could be risk factors for in-hospital morbidity and mortality [27,28]. Thus, the association between hyperglycemia and hospital outcomes among patients with T2D might in part be attenuated by the fact that T2D patients without hyperglycemia are more likely to have less favorable baseline characteristics compared with T2D patients with hyperglycemia.

We found in the univariate analysis that acute kidney injury was more common in T2D patients with poor diabetes control, as reflected by HbA1C ≥ 7%. However, the association become insignificant after adjusting for the presence of underlying CKD. This indicates that the observed association is likely mediated by decreased baseline kidney function in patients with poor diabetes control. We also found in the univariate analysis that metformin use and sulfonylurea use were associated with decreased mortality. Although the sample size was relatively low and this observation can be confounded by indication, our finding is consistent with a recent meta-analysis showing the association between metformin use and lower mortality (pooled adjusted OR 0.64, 95%CI 0.43–0.97), which is thought to be secondary to the pleiotropic immunomodulatory effects of metformin that result in lower levels of circulating pro-inflammatory cytokines [29]. On the other hand, an association between sulfonylurea use and COVID-19 outcome has not been reported in other studies; further studies need to be carried out to explore and validate this finding. We did not find an association between dipeptidyl peptidase-4 inhibitor and mortality, as has been observed in some other observational studies [30,31,32]. Due to the limited sample size, we do not have enough information to determine the association between hospital outcomes and the use of sodium-glucose cotransporter-2 inhibitors, which has been suggested to increase the likelihood of COVID-19-related ketoacidosis among patients with severe insulin deficiency [32]. 

There are certain limitations that should be acknowledged when interpreting the results of this study. First, this is a retrospective study; therefore, there are inherent limitations in the available information and/or documentation (e.g., comorbidities, hospital outcomes, and laboratory data). Second, the number of patients without T2D who had hyperglycemia upon admission was relatively small, which may result in limited statistical power. Third, the data of COVID-19 patients hospitalized between March and August 2020 were used in this study. Thus, the treatment strategy in our study may be different from the most updated standard treatment for COVID-19 [33]. Furthermore, as we recorded the most recent HbA1C within 2 years of admission and only 27% of the patients had HbA1C levels available within 3 months prior to admission, the HbA1C levels may not accurately represent glycemic control in patients with T2D at the time of hospitalization. Additionally, since a significant number of patients visiting our institution were uninsured and did not have access to long-term medical care, some hyperglycemic patients without a history of T2D may have had occult metabolic disorders that were left undiagnosed and untreated. Therefore, occult comorbidities could be one of the unmeasured confounders. However, in our cohort, no difference in in-hospital mortality was observed between patients with T2D based on diabetes control prior to admission as assessed by HbA1c, implying that the effect of chronically uncontrolled diabetes may play a less significant role in determining the risk of severity of COVID-19 than BG admission, when considered as a marker of level of illness. Finally, as our study centered on only hospitalized COVID-19 patients, the result may not be generalizable to non-hospitalized patients. 

## 5. Conclusions

We demonstrated a significant association between hyperglycemia upon admission and increased hospital mortality and morbidity in COVID-19 patients with and without T2D, and the association was stronger among patients without T2D. Additionally, patients without T2D who presented with hyperglycemia seemed to have more severe clinical outcomes compared with those with T2D, even after adjusting for admission BG and comorbidities. The result may be explained by considering stress hyperglycemia to be a marker of higher inflammatory burden and illness severity. Moreover, the direct cytopathic effect of SARS-CoV-2 on pancreatic β-cells, resulting in β-cell dysfunction and insulin deficiency, may play a role.

## Figures and Tables

**Table 1:** Characteristics of patients with and without type 2 diabetes who had blood glucose >200 mg/dL and ≤200 mg/dL at hospital presentation.

Characteristics	Patients without Type 2 Diabetes			Patients with Type 2 Diabetes		
	Blood Glucose > 200 mg/dL (*N* = 42)	Blood Glucose ≤ 200 mg/dL (*N* = 934)	*p*-Value	Blood Glucose > 200 mg/dL (*N* = 193)	Blood Glucose ≤ 200 mg/dL (*N* = 265)	*p*-Value
Age (years old)	57.0 (46.5–69.0)	52.0 (38.0–64.25)	0.055	63.0 (53.0–72.0)	65.0 (57.0–74.0)	0.065
Female sex	17 (40.5%)	409 (43.8%)	0.672	79 (40.9%)	137 (51.7%)	0.023 *
BMI (kg/m^2^)	28.9 (24.1–32.7)	28.5 (24.5–33.9)	0.511	28.8 (25.1–33.0)	29.9 (25.2–35.6)	0.156
Race						
White	24 (57.1%)	506 (54.2%)	0.439	81 (42.0%	109 (41.1%)	0.974
Black	15 (35.7%)	393 (42.1%)		106 (54.9%)	147 (55.5%)	
Other	3 (7.1%)	35 (3.7%)		6 (3.1%)	9 (3.4%)	
History of smoking	13 (31.0%)	317 (33.9%)	0.869	72 (37.3%)	132 (49.8%)	0.008 *
Alcohol use	6 (14.3%)	299 (32.0%)	0.015^*^	53 (27.5%)	87 (32.8%)	0.218
Control of diabetes						
HbA1C < 7%	-	-	-	133 (68.9%)	217 (81.9%)	0.005 *
HbA1C 7– < 8%	-	-	-	16 (8.3%)	20 (7.5%)	
HbA1C 8– < 9%	-	-	-	11 (5.7%)	8 (3.0%)	
HbA1C 9– < 10%	-	-	-	8 (4.1%)	8 (3.0%)	
HbA1C ≥ 10%	-	-	-	25 (13.0%)	12 (4.5%)	
Antidiabetic medications						
Metformin	-	-	-	91 (47.2%)	102 (38.5%)	0.064
DPP-4 inhibitors	-	-	-	28 (14.5%)	35 (13.2%)	0.690
SGLT-2 inhibitors	-	-	-	9 (4.7%)	9 (3.4%)	0.491
Sulfonylureas	-	-	-	49 (25.4%)	36 (13.6%)	0.001 *
GLP-1 agonists	-	-	-	29 (15.0%)	29 (10.9%)	0.195
Insulin				129 (66.8%)	161 (60.8%)	0.182
Underlying diseases						
Hypertension	13 (31.0%)	361 (38.7%)	0.315	158 (81.9%)	232 (87.5%)	0.091
Dyslipidemia	7 (16.7%)	205 (21.9%)	0.417	131 (67.9%)	178 (67.2%)	0.874
Coronary heart disease	2 (4.8%)	47 (5.0%)	1.000	21 (10.9%)	43 (16.2%)	0.103
Heart failure	2 (4.8%)	63 (6.7%)	0.614	19 (9.8%)	60 (22.6%)	<0.001 *
Cerebrovascular disease	0 (0.0%)	27 (2.9%)	0.625	5 (2.6%)	27 (10.2%)	0.001 *
Asthma	4 (9.5%)	119 (12.7%)	0.811	27 (14.0%)	54 (20.4%)	0.077
COPD	2 (4.8%)	61 (6.5%)	1.000	7 (3.6%)	33 (12.5%)	0.001 *
CKD	0 (0.0%)	76 (8.1%)	0.069	46 (23.8%)	93 (35.1%)	0.010 *
ESRD	0 (0.0%)	27 (2.9%)	0.264	5 (2.6%)	35 (13.2%)	<0.001 *
Malignancy	3 (7.1%)	80 (8.6%)	1.000	20 (10.4%)	46 (17.4%)	0.035 *
HIV infection	2 (4.8%)	30 (3.2%)	0.644	4 (2.1%)	6 (2.3%)	0.890
In-hospital medical therapy for COVID-19						
Azithromycin	11 (26.2%)	365 (39.1%)	0.093	91 (47.2%)	129 (48.7%)	0.746
Colchicine	6 (14.3%)	136 (14.6%)	0.961	32 (16.6%)	44 (16.6%)	0.955
Hydroxychloroquine	11 (26.2%)	425 (45.5%)	0.014 *	106 (54.9%)	140 (52.8%)	0.657
Corticosteroids	9 (21.4%)	77 (8.2%)	0.003 *	23 (11.9%)	25 (9.4%)	0.392
IL-6 antibodies	6 (14.3%)	172 (18.4%)	0.498	50 (25.9%)	56 (21.1%)	0.232
IL-1 receptor antagonists	1 (2.4%)	30 (3.2%)	1.000	5 (2.6%)	11 (4.2%)	0.446
Remdesivir	6 (14.3%)	49 (5.2%)	0.013 *	8 (4.1%)	8 (3.0%)	0.517

Note: “*” denotes *p* < 0.05. Data are expressed as median (interquartile range) or number of patients (%). Abbreviations: BMI: Body Mass Index; COPD: Chronic Obstructive Pulmonary Disease; CKD: Chronic Kidney Disease; DPP-4: Dipeptidyl Peptidase-4; ESRD: End-Stage Renal Disease; GLP-1; Glucagon-Like Peptide-1; HbA1C: Hemoglobin A1C; HIV: Human Immunodeficiency Virus; SGLT-2; Sodium-Glucose Cotransporter-2.

**Table 2:** Univariate comparisons of hospital outcomes between patients with admission blood glucose >200 mg/dL and ≤200 mg/dL among patients with and without type 2 diabetes.

Hospital Outcomes	Patients without Type 2 diabetes			Patients with Type 2 Diabetes		
	Blood Glucose > 200 mg/dL (*N* = 42)	Blood Glucose ≤ 200 mg/dL (*N* = 934)	*p*-Value	Blood Glucose > 200 mg/dL (*N* = 193)	Blood Glucose ≤ 200 mg/dL (*N* = 265)	*p*-Value
Death	9 (21.4%)	62 (6.6%)	<0.001 *	25 (13.0%)	36 (13.6%)	0.844
ICU admission	16 (40.0%)	151 (16.2%)	<0.001 *	48 (24.9%)	54 (20.4%)	0.254
Intubation	15 (35.7%)	100 (10.7%)	<0.001 *	34 (17.6%)	36 (13.6%)	0.236
Hospital length of stay (days)	10.1 ± 16.8	7.1 ± 8.3	0.036 *	9.4 ± 11.6	8.5 ± 9.0	1.000
ARDS	6 (14.3%)	70 (7.5%)	0.108	25 (13.0%)	23 (8.7%)	0.140
Myocardial infarction	3 (7.1%)	42 (4.5%)	0.437	17 (8.8%)	21 (7.9%)	0.735
Acute kidney injury	17 (40.5%)	219 (23.4%)	0.012 *	104 (53.9%)	96 (36.2%)	<0.001 *
Severe sepsis/septic shock	10 (23.8%)	73 (7.8%)	<0.001 *	26 (13.5%)	40 (15.1%)	0.625

“*” denotes *p* < 0.05. Data are expressed as mean ± SD or number of patients (%). Abbreviation: ARDS: Acute Respiratory Distress Syndrome; ICU: Intensive Care Unit.

**Table 3:** Adjusted association between admission blood glucose >200 mg/dL and hospital outcomes in patients with and without type 2 diabetes.

Hospital Outcomes	Patients without Type 2 Diabetes			Patients with Type 2 Diabetes		
	Adjusted OR	95% CI	*p*-Value	Adjusted OR	95% CI	*p*-Value
Death	4.46	1.77–11.20	0.001 *	1.55	0.81–2.98	0.184
ICU admission	3.71	1.84–7.48	<0.001 *	1.69	1.03–2.79	0.039 *
Intubation	4.82	2.39–9.72	<0.001 *	1.68	0.94–2.98	0.078
ARDS	1.92	0.75–4.91	0.176	1.81	0.93–3.53	0.081
Myocardial infarction	1.69	0.47–6.03	0.421	1.55	0.73–3.27	0.252
Acute kidney injury	2.29	1.14–4.60	0.020 *	2.45	1.54–3.90	<0.001 *
Severe sepsis/septic shock	4.14	1.85–9.24	<0.001 *	1.05	0.58–1.88	0.882

“*” denotes *p* < 0.05. Reference groups were patients with admission blood glucose ≤200 mg/dL. Effect estimates were adjusted for age; sex; body mass index; smoking; alcohol use; and underlying comorbidities, including hypertension, dyslipidemia, coronary heart disease, cerebrovascular disease, chronic obstructive pulmonary disease, asthma, chronic kidney disease, end-stage renal disease, malignancy, human immunodeficiency virus infection, and heart failure. Abbreviations: ARDS: Acute Respiratory Distress Syndrome; ICU: Intensive Care Unit.

**Table 4:** Comparison of hospital outcomes between patients with and without type 2 diabetes among patients with admission blood glucose >200 mg/dL.

Hospital Outcomes	Univariate Analysis			Multivariate Analysis		
	Patients with Type 2 Diabetes (*N* = 42)	Patients without Type 2 Diabetes (*N* = 193)	*p*-Value	Adjusted OR	95% CI	*p*-Value
Death	25 (13.0%)	9 (21.4%)	0.157	5.05	1.34–18.99	0.016 *
ICU admission	48 (24.9%)	16 (40.0%)	0.051	2.68	1.07–6.71	0.035 *
Intubation	34 (17.6%)	15 (35.7%)	0.009 *	3.78	1.37–10.46	0.010 *
ARDS	25 (13.0%)	6 (14.3%)	0.817	1.44	0.42–4.94	0.567
Myocardial infarction	17 (8.8%)	3 (7.1%)	1.000	0.61	0.11–3.31	0.570
Acute kidney injury	104 (53.9%)	17 (40.5%)	0.115	1.24	0.49–3.15	0.649
Severe sepsis/septic shock	26 (13.5%)	10 (23.8%)	0.092	3.90	1.28–11.99	0.017 *

“*” denotes *p* <0.05. Reference group was patients with diabetes and admission blood glucose >200 mg/dL. Effect estimates were adjusted for admission blood glucose; age; sex; body mass index; smoking; alcohol use; and underlying comorbidities, including hypertension, dyslipidemia, coronary heart disease, cerebrovascular disease, chronic obstructive pulmonary disease, asthma, chronic kidney disease, end-stage renal disease, malignancy, human immunodeficiency virus infection, and heart failure. Abbreviation: ARDS: Acute Respiratory Distress Syndrome; ICU: Intensive Care Unit.

## Data Availability

Data will be available upon reasonable request to mfholick@bu.edu.

## Supplementary Materials

The following are available online at https://www.mdpi.com/article/10.3390/nu13072199/s1: Table S1: univariate association of long-term diabetes control and use of anti-diabetic medications with hospital outcomes among patients with type 2 diabetes.

## References

[1] Hu B., Guo H., Zhou P., Shi Z.-L. (2020). Characteristics of SARS-CoV-2 and COVID-19. Nat. Rev. Microbiol..

[2] The Lancet Infectious Disease (2020). COVID-19, a pandemic or not?. Lancet Infect. Dis..

[3] Kordzadeh-Kermani E., Khalili H., Karimzadeh I. (2020). Pathogenesis, clinical manifestations and complications of coronavirus disease 2019 (COVID-19). Future Microbiol..

[4] Wiersinga W.J., Rhodes A., Cheng A.C., Peacock S.J., Prescott H.C. (2020). Pathophysiology, Transmission, Diagnosis, and Treatment of Coronavirus Disease 2019 (COVID-19): A Review. JAMA.

[5] Palaiodimos L., Chamorro-Pareja N., Karamanis D., Li W., Zavras P.D., Chang K.M., Mathias P., Kokkinidis D.G. (2020). Diabetes is associated with increased risk for in-hospital mortality in patients with COVID-19: A systematic review and meta-analysis comprising 18,506 patients. Hormones.

[6] Roncon L., Zuin M., Rigatelli G., Zuliani G. (2020). Diabetic patients with COVID-19 infection are at higher risk of ICU admission and poor short-term outcome. J. Clin. Virol..

[7] Shang L., Shao M., Guo Q., Shi J., Zhao Y., Xiaokereti J., Tang B. (2020). Diabetes Mellitus is Associated with Severe Infection and Mortality in Patients with COVID-19: A Systematic Review and Meta-analysis. Arch. Med. Res..

[8] Roberts J., Pritchard A.L., Treweeke A.T., Rossi A.G., Brace N., Cahill P., MacRury S.M., Wei J., Megson I.L. (2021). Why Is COVID-19 More Severe in Patients with Diabetes? The Role of Angiotensin-Converting Enzyme 2, Endothelial Dysfunction and the Immunoinflammatory System. Front. Cardiovasc. Med..

[9] Muniangi-Muhitu H., Akalestou E., Salem V., Misra S., Oliver N.S., Rutter G.A. (2020). Covid-19 and Diabetes: A Complex Bidirectional Relationship. Front. Endocrinol..

[10] Kusmartseva I., Wu W., Syed F., Van Der Heide V., Jorgensen M., Joseph P., Tang X., Candelario-Jalil E., Yang C., Nick H. (2020). Expression of SARS-CoV-2 Entry Factors in the Pancreas of Normal Organ Donors and Individuals with COVID-19. Cell Metab..

[11] Mazori A.Y., Bass I.R., Chan L., Mathews K.S., Altman D.R., Saha A., Soh H., Wen H.H., Bose S., Leven E. (2021). Hyperglycemia is Associated with Increased Mortality in Critically Ill Patients with COVID-19. Endocr. Pract..

[12] Fadini G.P., Morieri M.L., Boscari F., Fioretto P., Maran A., Busetto L., Bonora B.M., Selmin E., Arcidiacono G., Pinelli S. (2020). Newly-diagnosed diabetes and admission hyperglycemia predict COVID-19 severity by aggravating respiratory deterioration. Diabetes Res. Clin. Pract..

[13] Yang Y., Cai Z., Zhang J. (2021). Hyperglycemia at admission is a strong predictor of mortality and severe/critical complications in COVID-19 patients: A meta-analysis. Biosci. Rep..

[14] Prattichizzo F., Giuliani A., Sabbatinelli J., Matacchione G., Ramini D., Bonfigli A.R., Rippo M.R., de Candia P., Procopio A.D., Olivieri F. (2020). Prevalence of residual inflammatory risk and associated clinical variables in patients with type 2 diabetes. Diabetes Obes. Metab..

[15] Sachdeva S., Desai R., Gupta U., Prakash A., Jain A., Aggarwal A. (2020). Admission Hyperglycemia in Non-diabetics Predicts Mortality and Disease Severity in COVID-19: A Pooled Analysis and Meta-summary of Literature. SN Compr. Clin. Med..

[16] Godinjak A., Iglica A., Burekovic A., Jusufovic S., Ajanovic A., Tancica I., Kukuljac A. (2015). Hyperglycemia in Critically Ill Patients: Management and Prognosis. Med. Arch..

[17] Palermo N.E., Gianchandani R.Y., McDonnell M.E., Alexanian S.M. (2016). Stress Hyperglycemia During Surgery and Anesthesia: Pathogenesis and Clinical Implications. Curr. Diabetes Rep..

[18] Dungan K.M., Braithwaite S.S., Preiser J.-C. (2009). Stress hyperglycaemia. Lancet.

[19] Yu W.-K., Li W.-Q., Li N., Li J.-S. (2003). Influence of acute hyperglycemia in human sepsis on inflammatory cytokine and counterregulatory hormone concentrations. World J. Gastroenterol..

[20] Stentz F.B., Umpierrez G.E., Cuervo R., Kitabchi A.E. (2004). Proinflammatory Cytokines, Markers of Cardiovascular Risks, Oxidative Stress, and Lipid Peroxidation in Patients with Hyperglycemic Crises. Diabetes.

[21] Boddu S.K., Aurangabadkar G., Kuchay M.S. (2020). New onset diabetes, type 1 diabetes and COVID-19. Diabetes Metab. Syndr..

[22] Nassar M., Nso N., Baraka B., Alfishawy M., Mohamed M., Nyabera A., Sachmechi I. (2021). The association between COVID-19 and type 1 diabetes mellitus: A systematic review. Diabetes Metab. Syndr..

[23] Rubino F., Amiel S.A., Zimmet P., Alberti G., Bornstein S., Eckel R.H., Mingrone G., Boehm B., Cooper M.E., Chai Z. (2020). New-Onset Diabetes in Covid-19. N. Engl. J. Med..

[24] Sardu C., D’Onofrio N., Balestrieri M.L., Barbieri M., Rizzo M.R., Messina V., Maggi P., Coppola N., Paolisso G., Marfella R. (2020). Outcomes in Patients with Hyperglycemia Affected by COVID-19: Can We Do More on Glycemic Control?. Diabetes Care.

[25] Marfella R., Sasso F.C., Cacciapuoti F., Portoghese M., Rizzo M.R., Siniscalchi M., Carbonara O., Ferraraccio F., Torella M., Petrella A. (2012). Tight Glycemic Control May Increase Regenerative Potential of Myocardium during Acute Infarction. J. Clin. Endocrinol. Metab..

[26] Sasso F.C., Rinaldi L., Lascar N., Marrone A., Pafundi P.C., Adinolfi L.E., Marfella R. (2018). Role of Tight Glycemic Control during Acute Coronary Syndrome on CV Outcome in Type 2 Diabetes. J. Diabetes Res..

[27] Corrao S., Santalucia P., Argano C., Djade C.D., Barone E., Tettamanti M., Pasina L., Franchi C., Kamal Eldin T., Marengoni A. (2014). Gender-differences in disease distribution and outcome in hospitalized elderly: Data from the REPOSI study. Eur. J. Intern. Med..

[28] Marcucci M., Franchi C., Nobili A., Mannucci P.M., Ardoino I., Investigators R. (2017). Defining Aging Phenotypes and Related Outcomes: Clues to Recognize Frailty in Hospitalized Older Patients. J. Gerontol. Ser. A.

[29] Prattichizzo F., Sabbatinelli J., de Candia P., Olivieri F., Ceriello A. (2021). Tackling the pillars of ageing to fight COVID-19. Lancet Healthy Longev..

[30] Pal R., Banerjee M., Mukherjee S., Bhogal R.S., Kaur A., Bhadada S.K. (2021). Dipeptidyl peptidase-4 inhibitor use and mortality in COVID-19 patients with diabetes mellitus: An updated systematic review and meta-analysis. Ther. Adv. Endocrinol. Metab..

[31] Solerte S.B., D’Addio F., Trevisan R., Lovati E., Rossi A., Pastore I., Dell’Acqua M., Ippolito E., Scaranna C., Bellante R. (2020). Sitagliptin Treatment at the Time of Hospitalization Was Associated with Reduced Mortality in Patients with Type 2 Diabetes and COVID-19: A Multicenter, Case-Control, Retrospective, Observational Study. Diabetes Care.

[32] Mirabelli M., Chiefari E., Puccio L., Foti D.P., Brunetti A. (2020). Potential Benefits and Harms of Novel Antidiabetic Drugs During COVID-19 Crisis. Int. J. Environ. Res. Public Health.

[33] Umakanthan S., Chattu V.K., Ranade A.V., Das D., Basavarajegowda A., Bukelo M. (2021). A rapid review of recent advances in diagnosis, treatment and vaccination for COVID-19. AIMS Public Health.

