# Supplementary material for: Association between Hyperglycemia at Hospital Presentation and Hospital Outcomes in COVID-19 Patients with and without Type 2 Diabetes: A Retrospective Cohort Study of Hospitalized Inner-City COVID-19 Patients
Source: Nutrients. 2021 Jun 26;13(7):2199. doi: 10.3390/nu13072199 (PMC8308462; doi:10.3390/nu13072199)
Supplement: Supplementary file 1 [file nutrients-13-02199-s001.zip › nutrients-1206373-supplementary.pdf]

nutrients

Supplementary Materials

Association between Hyperglycemia at Hospital Presentation and Hospital Outcomes in COVID-19 Patients with and without Type 2 Diabetes: A Retrospective Cohort Study of Hospitalized Inner-City COVID-19 Patients

Nipith Charoenngam [1,2], Sara M. Alexanian [1], Caroline M. Apovian [3] and Michael F. Holick [1,*]

Table S1. Univariate association of long-term diabetes control and use of anti-diabetic medications with hospital outcomes among patients with type 2 diabetes (*N* = 458).

	**Death**	***P*-value**	**ICU admission**	***P*-value**	**Intubation**	***P*-value**	**ARDS**	***P*-value**	**Myocardial infarction**	***P*-value**	**Acute kidney injury**	***P*-value**	**Severe sepsis/septic shock**	***P*-value**
							Long-term glycemic control							
HbA1C <7% (*N* = 350)	14 (13.0%)	0.901	24 (22.2%)	0.989	15 (13.9%)	0.645	10 (9.3%)	0.636	11 (10.2%)	0.416	56 (51.9%)	0.050	16 (14.8%)	0.891
HbA1C ≥7% (*N* = 108)	47 (13.4%)		78 (22.3%)		55 (15.7%)		38 (10.9%)		27 (7.7%)		144 (41.1%)		50 (14.3%)	
							Use of anti-diabetic medications							
Metformin (*N* = 193)	18 (9.3%)	0.032	35 (18.1%)	0.069	19 (9.8%)	0.006	16 (9.3%)	0.192	11 (5.7%)	0.085	85 (44.0%)	0.891	21 (10.9%)	0.066
No metformin (*N* = 265)	43 (16.2%)		67 (25.3%)		51 (19.2%)		32 (12.1%)		27 (10.2%)		115 (43.4%)		45 (17.0%)	
DPP-4 inhibitors (*N* = 63)	8 (12.7%)	0.876	16 (25.4%)	0.521	9 (14.3%)	0.813	4 (6.3%)	0.374	6 (9.5%)	0.704	38 (60.3%)	0.004	9 (14.3%)	0.976
No DPP-4 inhibitors (*N* = 395)	53 (13.4%)		86 (21.8%)		61 (15.4%)		44 (11.1%)		32 (8.1%)		162 (41.0%)		57 (14.4%)	
SGLT-2 inhibitors (*N* = 440)	0 (0.0%)	0.149	4 (22.2%)	1.000	2 (11.1%)	1.000	1 (5.6%)	0.708	2 (11.1%)	0.654	11 (61.1%)	0.128	2 (11.1%)	0.684
No SGLT-2 Inhibitors (*N* = 18)	61 (13.9%)		98 (22.3%)		68 (15.5%)		47 (10.7%)		36 (8.2%)		189 (43.0%)		64 (14.5%)	
Sulfonylureas (*N* = 373)	4 (4.7%)	0.010	18 (21.2%)	0.788	11 (12.9%)	0.506	5 (5.9%)	0.125	7 (8.2%)	0.982	44 (51.8%)	0.095	7 (8.2%)	0.072

No sulfonylureas (*N* = 85)	57 (15.3%)		84 (22.5%)		59 (15.8%)		43 (11.5%)		31 (8.3%)		156 (41.8%)		59 (15.8%)	
GLP-1 agonists (*N* = 58)	7 (12.1%)	0.764	15 (25.9%)	0.482	10 (17.2%)	0.658	8 (13.8%)	0.378	6 (10.3%)	0.545	29 (50.0%)	0.298	8 (13.8%)	0.886
No GLP-1 agonists (*N* = 400)	54 (13.5%)		87 (21.8%)		60 (15.0%)		40 (10.0%)		32 (8.0%)		171 (42.8%)		58 (14.5%)	
Insulin (*N* = 290)	43 (14.8%)	0.212	73 (25.2%)	0.50	44 (15.2%)	0.931	28 (9.7%)	0.449	29 (10.0%)	0.083	142 (49.0%)	0.003	48 (16.6%)	0.086
No insulin (*N* = 168)	18 (10.7%)		29 (17.3%)		26 (15.5%)		20 (11.9%)		9 (5.4%)		38 (34.5%)		18 (10.7%)	

Abbreviations: DPP-4: Dipeptidyl Peptidase-4; GLP-1; Glucagon-like Peptide-1; HbA1C: Hemoglobin A1C; SGLT-2; Sodium-glucose Cotransporter-2.
